# Functional Neuroimaging in Dissociative Disorders: A Systematic Review

**DOI:** 10.3390/jpm12091405

**Published:** 2022-08-29

**Authors:** Martina Nicole Modesti, Ludovica Rapisarda, Gabriela Capriotti, Antonio Del Casale

**Affiliations:** 1 Faculty of Medicine and Psychology, Sapienza University of Rome, Via di Grottarossa 1035, 00189 Rome, Italy; 2 Unit of Nuclear Medicine, Department of Medical-Surgical Sciences and Translational Medicine, Faculty of Medicine and Psychology, Sant’Andrea University Hospital, Sapienza University of Rome, Via di Grottarossa 1035, 00189 Rome, Italy; 3 Unit of Psychiatry, Department of Dynamic and Clinical Psychology, Faculty of Medicine and Psychology, Sant’Andrea University Hospital, Sapienza University of Rome, Via di Grottarossa 1035, 00189 Rome, Italy

**Keywords:** dissociative disorders, dissociative identity disorder, depersonalization disorder, dissociative amnesia, fMRI, PET, functional neuroimaging

## Abstract

Background: Dissociative disorders encompass loss of integration in essential functions such as memory, consciousness, perception, motor control, and identity. Nevertheless, neuroimaging studies, albeit scarce, have suggested the existence of particular brain activation patterns in patients belonging to this diagnostic category. The aim of this review is to identify the main functional neuroimaging correlates of dissociative disorders. Methods: we searched the PubMed database to identify functional neuroimaging studies conducted on subjects with a diagnosis of a dissociative disorder, following the PRISMA guidelines. In the end, we included 13 studies in this systematic review, conducted on 51 patients with dissociative identity disorder (DID), 28 subjects affected by depersonalization disorder, 24 with dissociative amnesia, and 6 with other or not specified dissociative disorders. Results: Prefrontal cortex dysfunction seems prominent. In addition, changes in the functional neural network of the caudate are related to alterations of identity state and maintenance of an altered mental status in DID. Another role in DID seems to be played by a dysfunction of the anterior cingulate gyrus. Other regions, including parietal, temporal, and insular cortices, and subcortical areas were reported to be dysfunctional in dissociative disorders. Conclusions: Prefrontal dysfunction is frequently reported in dissociative disorders. Functional changes in other cortical and subcortical areas can be correlated with these diagnoses. Further studies are needed to clarify the neurofunctional correlations of each dissociative disorder in affected patients, in order to identify better tailored treatments.

## 1. Introduction

Dissociation encompasses loss of integration in essential functions such as memory, consciousness, perception, motor control, and identity [1]. Although these are established conclusions, the classification of dissociative disorders has undergone many changes through the decades, being initially assimilated to what are nowadays called somatoform disorders, and that were once defined as neuroses. The DSM-II [2] included two categories of hysterical neurosis (conversion type and dissociative type) and a hysterical (histrionic) personality disorder, but the atheoretically-oriented DSM-III [3] omitted the term “neurosis” and initially conserved the term “hysterical” only in brackets, splitting hysterical neurosis conversion type into conversion disorder and psychogenic pain disorder in the context of somatoform disorders, and hysterical neurosis dissociative type into psychogenic amnesia, psychogenic fugue, and multiple personality disorder in the context of dissociative disorders (sleepwalking disorder was placed among childhood disorders). This trend was confirmed in the subsequent revisions up to DSM-IV-TR [4]. DSM-5 has maintained the Dissociative Disorders, somehow renaming the disorders included and adding new ones (Dissociative Identity Disorder, Dissociative Amnesia, Depersonalization/Derealization Disorder, Other Specified Dissociative Disorder, and Unspecified Dissociative Disorder). Dissociative identity disorder in DSM-5 now refers to possession and/or identity fragmentation, in order to be more aligned with possible cultural differences. Dissociative amnesia includes dissociative fugue as a subtype, since fugue is a rare disorder that always involves amnesia but does not always include confused wandering or loss of personality identity [5]. Depersonalization disorder also includes derealization, since the two often co-occur. As far as single symptoms are concerned, severe dissociation has been associated with childhood trauma exposure [6,7] and meta-analyses have proved that all forms of childhood traumas are directly associated with dissociation in patients with mental disorders [6]. In fact, patients affected by dissociative identity disorder (DID) report severe childhood traumas [8,9], while patients suffering from depersonalization disorder (DPD) report lower childhood trauma exposure, also compared to patients with depressive disorders [10,11]. It is unclear whether similar disorders with different severity may share neural correlates and etiology, but findings on neural alterations may help solve this puzzle.

In the last four decades, neuroimaging studies have focused on dissociative disorders, suggesting the existence of particular brain activation patterns in patients belonging to this diagnostic category. These studies have shown regional cerebral blood flow (rCBF) or glucose metabolism (CMRglu) changes in the temporal, parietal (implicated in dissociative and conversion phenomena), and orbitofrontal (likely involved in the integration of the self) cortices, in the hippocampus (involved in memory and in the removal of traumatic events), in the basal ganglia, and in other areas. However, they have reached different, and at times conflicting, conclusions, finding out hyperactivation of brain areas in some cases [12,13,14], and hypoactivation in others [15]. It is also unclear whether these changes involve more often the left or right hemisphere, albeit recent evidence suggests greater damage of the former. Furthermore, most of these studies are limited by low sample size, heterogeneity of diagnosis and comorbidity, and stage of disease (onset, acute, or chronic).

Established models have linked dissociation with increased recruitment in regions involved in executive control [16,17], such as the ventromedial prefrontal cortex, anterior cingulate cortex, and inferior frontal gyri, resulting in dampened activation of the amygdala. Attention has also been focused on the connectivity of temporal cortices, specifically the temporoparietal junctions, because of its involvement in the neurophysiology of multisensory integration [18], whose processing can be altered in case of out of body experiences during brain stimulation and lesion studies [19,20].

We hypothesized that there could be common functional changes in patients with a DSM diagnosis of dissociative disorders across different brain regions during task execution and/or during resting state, and that these alterations would match impaired performance of neurocognitive tasks. Considering the low prevalence of dissociative disorders, the low number of functional neuroimaging studies and their mostly inconsistent results, we aimed to clarify the main neural functional pattern associated with these diagnoses. This review aimed to identify alterations in functional neuroimaging techniques reports, and to correlate them with impaired neurocognitive performance and psychopathology of patients with DDs.

## 2. Materials and Methods

A search was conducted using the international scientific database PubMed (http://www.pubmed.gov; accessed on 2 August 2022) to identify functional neuroimaging studies conducted on subjects with a diagnosis of dissociative disorder (i.e., dissociative identity disorder, depersonalization disorder, dissociative amnesia, and other or not specified dissociative disorders). We followed the methods of the Preferred Reporting Items for Systematic Reviews and Meta-Analyses (PRISMA Statement) [21]. First, we searched the database PubMed with the title/abstract filter, using the keywords “dissociative disord*”, “dissociative amnesia”, “identity disorder”, “PET”, “MRI”, “positron emission”, “functional magnetic”, and “spectroscopy.” Second, we collected further studies by examining the bibliographies of relevant articles in the first step or through the “related article” function of the PubMed database, and bibliographies of reviews on the topic.

We included articles on functional neuroimaging studies in samples of patients diagnosed with dissociative disorders (Dissociative Identity Disorder, Dissociative Amnesia, Depersonalization/Derealization Disorder, Other Specified Dissociative Disorder, and Unspecified Dissociative Disorder) according to the DSM criteria, regardless of the presence of healthy controls in the study. We excluded case reports, case studies, pharmacological trials, reviews, studies on children, studies employing techniques other than functional neuroimaging, and studies involving patients with other psychiatric diagnoses. Three reviewers screened each record and each report reviewed, and collected reported data, working independently from each other, and subsequently verifying together the appropriateness of the procedure, and proofreading the included data and results. Two reviewers independently assessed the risk of bias in each included study, considering the sample number, the inclusion/exclusion criteria of participants, the consistency of the sample, and the methods employed, and used the Scottish Intercollegiate Guidelines Network (SIGN) quality assessment tool [22]. We did not register and prepare this study on the basis of a pre-established protocol. We did not use automation tools in our methodological processes.

Based on these criteria, we analyzed 61 studies, from which we excluded 48 studies, of which 26 were unrelated to the subject, 6 were case reports/case studies [15,23,24,25,26,27], 1 was focused on schizophrenia [28], 1 was conducted on adolescents with post-traumatic stress disorder [29], 1 was conducted on healthy subjects [30], 7 used structural neuroimaging techniques [31,32,33,34,35,36,37], and 3 were not focused on dissociative disorders [38,39,40]. We finally included 13 studies published between 2006 and 2022 [14,33,34,41,42,43,44,45,46,47,48,49,50]. We grouped the studies in our discussion on the basis of the most consistently reported dysfunctional brain areas in affected patients.

The main study outcomes for which data were sought were changes in neural cortical and subcortical activation patterns and changes in neural metabolism correlated with the diagnosis of a dissociative disorder and/or with symptom severity in patients with a dissociative disorder.

### Quality Assessment Tool and Risk of Bias

The potential methodological quality of the study and risk of bias were examined through the SIGN quality assessment tool [22]. All studies addressed an appropriate and clearly focused question. Two studies had a high risk of bias in terms of study subjects, because they included two different diagnoses in the sample (view Table 1). In all 13 studies, nevertheless, the two groups being analyzed are selected from source populations that are comparable in all respects other than the factor under investigation. Even when rare differences were present (i.e., age), they were not statistically significant. There were no dropouts in the studies assessed, but no study specified how many of the people asked to take part did so, in each of the groups being studied. The outcomes are clearly defined in every study, the method of assessment of diagnosis is very reliable in each case (DSM), and all psychiatric diagnoses are established through these criteria worldwide. Confidence intervals have always been provided when analyzing brain functional changes in subjects. In conclusion, the biggest risk of bias lies only in the low sample of the studies, which could exaggerate the significance of the results [51]; nevertheless, given the low incidence of dissociative disorders in the general population, the results can be considered reliable. Taking into account clinical considerations, the evaluation of the methodology used, and the statistical power of the study, there is evidence of association between the disease and certain brain functional changes. In addition, according to the Grading of Recommendations Assessment, Development, and Evaluation (GRADE) system of rating the quality of evidence and grading strength of recommendations in systematic reviews [52], also taking into considerations the two aforementioned studies (view Table 1) which included two different diagnoses in the subject group, and therefore have a higher risk of selection bias, all included studies show consistent findings with no publication bias, no imprecisions, no serious limitations, and no serious indirectness. Ultimately, considering all the studies, their appropriateness of diagnosis as well as appropriate control of confounding, other aspects of design, conduct, and analysis that influence the risk of bias, we can conclude that the quality of evidence is high. Ideally, future systematic reviews on this topic will comprehensively summarize evidence on all important functional brain changes in dissociative disorders.

## 3. Results

**Figure 1:**
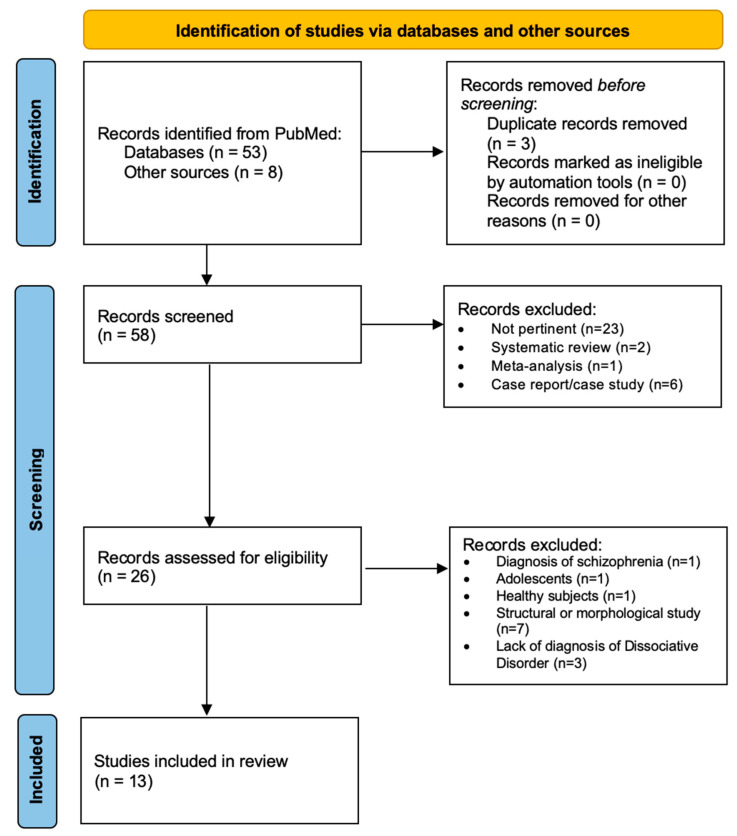
PRISMA search strategy on functional neuroimaging studies in dissociative disorders.

We summarized the search procedure in Figure 1, the main results in Table 1 below, and the PRISMA 2020 checklist in the Supplementary Table S1.

## 4. Discussion

### 4.1. Functional Changes in the Limbic/Paralimbic System

The most prominent functional changes have been found across the limbic system of subjects, mostly when they performed particular tasks whose execution may be affected by the disease.

Different functional neuroimaging studies were based on the Theory of Structural Dissociation of the Personality in DID, according to which patients retain traumatic memories fixed in the emotional part (EP), while some personality parts are apparently normal (ANP), in order to mentally avoid traumatic memories [41]. The EP parts of DID patients, when showed human faces expressing certain emotions (neutrality and anger), had a hyperactivation of the right parahippocampal gyrus, in comparison with the ANP part and with the EP part of controls (female actresses). This result can be associated with the clinical characteristics of EP (fixation on traumatic memories and tendency to reactivate traumatic memories with specific triggers). Thus, it can correlate to the fact that the parahippocampal gyrus are physiologically involved in the retaining of autobiographic memory, specifically the right one [53,54]. The ANP part of DID patients had a whole-brain decreased activity when showed both neutral and angry faces. The insula showed metabolic changes in a PET study during traumatic re-evocation in a traumatic state of identity (TIS) in patients affected by DID [44], confirming the impaired response to emotions, interoception, and physiological response to alarm [55]. Indeed, the insula seems to hyperactivate during egocentric learning in these patients [46]. Nevertheless, some peculiar tasks seem to be retained, if not improved, in dissociative disorders. Women with dissociative amnesia or DID during an egocentric virtual maze learning task showed preserved attentional visuospatial mnemonic functioning compared with controls [46]. Notwithstanding, higher severity of the clinical picture directly correlated with better performance, and was accompanied with the hyperactivation of the cingulate gyrus and the precuneus [47] This is consistent with the findings of another study in which DPD patients exhibited greater activation of the anterior cingulate during self-related processing [45], leading to the hypothesis that egocentric learning and egocentric tasks may be better in dissociative disorders than in healthy controls, due to the fact that dissociative defenses have improved and worked in avoiding the development of PTSD [46], or more severe psychiatric disorders. In fact, the cingulate gyrus, right anterior, and middle cingulate cortex seem to hypoactivate in traumatic vs. neutral identity states, as well as during within-identity state assessments [34] in patients with DID.

DPD patients have also shown anomalies in the neural function of some parts of the paralimbic system. The right anterior cingulate cortex (ACC) hyperactivates during face processing (self vs. stranger), which has been interpreted as a correlate of conflict between the conscious and unconscious aspects of self-processing in DPD [48]. It has been hypothesized that DPD patients’ problem lies not in consciously recognizing their own face, but in doing it unconsciously [45,56], associating this phenomenon with the hyperactivation of the ACC for conflict detection and resolution, thus explaining the presence of anguish in patients in acute depersonalization states. In addition, DPD patients also showed reduced activation of the amygdala in response to faces expressing emotions [48], a finding that could be associated with a response to traumatic events, as had previously been hypothesized in other studies on the matter, particularly on PTSD [57,58,59]. Different activation patterns in the right hippocampus and left amygdala have been associated with DID [50] due to the role of the former in the Papez memory circuit and its involvement in autobiographical memory. A neurobiological model of DID speculates that acute stress can be associated with a shift from hippocampal involvement to caudate involvement [50], which will be explained later to hyperactivate during traumatic identity state condition (see section below on subcortical areas)

### 4.2. Functional Changes in Frontal and Prefrontal Cortices

Changes in frontal and prefrontal cortices are an important finding of the review, and they particularly correlate with specific tasks or states of identity explained below.

DID patients showed hyperactivation of the prefrontal cortex during resting state, particularly in the dorsomedial area, and other areas of the default mode network (DMN), in comparison with healthy controls (actors) [33]. This suggests that DID patients are more involved when they need to concentrate on self. The same happened during the neutral personality state in response to trauma-related vs. neutral words [50], such as if their hyperarousal state triggered by trauma re-evocation could be lowered by prefrontal activation. On the contrary, healthy controls showed hypoactivation of the middle frontal gyrus during resting state [33].

A PET study has shown reduced resting state metabolism of the right inferolateral prefrontal cortex in patients with autobiographical memory loss due to dissociative amnesia, compared with healthy controls [42]. This region plays many roles, being specifically involved in self-awareness [60], self-regulatory processes [61], emotion regulation [62], and higher order control mechanisms [63], including active memory retrieval.

During an identity traumatic state in patients affected by DID, a reduced activation of the ventromedial prefrontal cortex and left frontal pole has been observed at fMRI during an n-back working memory task [43]. In addition, reduced memory performance has been found in correlation with this reduced activation, together with a higher number of mistakes during the task. These results can be related with symptoms such as flashback, re-experiencing traumatic events, anxiety, and other symptoms of DID. In fact, another study has demonstrated that patients with DID in an identity traumatic state, as well as during within-identity state assessment, compared to a neutral identity state, show a hypoactivation of the frontal cortex [34]. On the other hand, instead, since trauma revival is mostly sensorimotor- and anxiety-associated, it could also be related to reduced mentalization capacity (prefrontal deactivation), considering the essential role of this area in related functions [64]. These dysfunctions in the frontal cortex in traumatic states of identity can also relate with the presence of a retracted field of consciousness in patients with DID [43].

Other prefrontal areas (left anterior and dorsolateral cortices) have been found to hyperactivate during verbal working memory tasks in patients with DID or not specified dissociative disorder, when compared to healthy controls, and the same seems to happen in the left parietal lobe [14]. The same conclusions also apply to DPD patients [47,49]. These functional changes at fMRI are consistent with a working memory performance improvement in the same group of patients, when exposed to neutral memories. This finding can correlate with a different style of cognitive processing that these patients develop along the way. It is important to underline that hyperactivation in these areas occurs in response to neutral memories, because, as we have already seen, in traumatic states of identity, the frontoparietal activation is reduced [43]. The opposite, i.e., hypoactivation in the inferior parietal lobe, happens when these patients are instead employed in egocentric tasks [46]. This finding needs further clarification regarding whether it depends on the task or is a diagnostic correlate.

Various studies have also confirmed the presence of neurofunctional changes in the prefrontal cortex of DPD patients. In fact, during face processing (self vs. stranger faces), these patients had hyperactivation of the bilateral medial prefrontal cortices (PFCs) and left middle frontal gyrus (MFG), as well as the right anterior cingulate cortex [45,49]. Severity of depersonalization directly correlated with the increased activation of medial PFCs. This is consistent with a deficit in implicit self-processing [45].

### 4.3. Functional Changes in Other Cortical Areas

In addition to what has already been underlined above, other scattered cortical areas seem to undergo changes in dissociative disorders.

The left occipito-temporal junction was hyperactivated in the EP parts of DID patients, compared to the EP of controls, correlating with the role of this part of the cortex in face-sensitivity [41]. In addition, the primary somatosensory cortex was hyperactivated in the EP of DID patients during resting state, together with motor related areas [33]. This finding may be due to the fact that the EP of DID patients is more resting-state concentrated on the self and somatosensory feelings, and therefore self-awareness, together with an increased tendency to engage in motor reactions, such as if they are in an alert state due to previous traumas. Functional changes in numerous other cortical areas have been demonstrated in the studies we included, indicating that dissociative phenomena are complex and require further clarification in their neurophysiopathological correlates.

### 4.4. Subcortical Functional Changes

Changes in subcortical activation patterns have been found in dissociative disorder cases, mostly in the thalamic regions.

Compared to the EP part of controls (female actresses), those of DID patients, when showed neutral human faces, had a hyperactivation in the brainstem area, in correlation with clinical aspects of DID, for example hyperarousal [41].

The ANP of DID patients, compared to the EP, showed bilateral thalamus hyperactivation during resting state, and the same happens in healthy controls simulating ANP compared to when they simulate EP [33]. This finding is also confirmed by Weniger et al. [46], who found that the thalamus hyperactivates in healthy controls with respect to females with dissociative disorders and childhood abuse history. Thalamic hyperactivation correlates with the first biological models of dissociation in trauma survivors, in which the thalamus plays a fundamental role. [65] Another explanation for this thalamic hyperactivation lies in dysfunctional fear extinction processes in response to traumas [66]. Another PET study has shown that during a trauma re-evocation in a TIS in patients affected by DID, metabolic changes of the bilateral caudate nucleus were found, thus probably correlating with the role of the latter in the neurophysiology of anxiety disorders, obsessive–compulsive disorders (OCD), and generally in fear processing dysfunction [67,68,69,70]. The hypoactivation of the caudate in dissociative disorders during egocentric tasks has been confirmed by fMRI findings [46], whilst DID patients’ caudate nucleus hyperactivates during traumatic identity state condition, in response to personal trauma scripts [34]. The latter can be explained by the fact that the dorsal striatum i.e., caudate nucleus is involved both in the switch between identity states and in the maintenance of the altered identity state [15,41,50,71].

### 4.5. Limitations

The main limitation of this systematic review are related to the small number of functional neuroimaging studies in dissociative disorders. Because of this we have focused on the entire spectrum of dissociative disorders, and for this reason the eligibility criteria were heterogeneous. Another limitation is the use of only one database (PubMed), although we performed a further search by examining the bibliographies of relevant articles. The results of our systematic review should be taken with caution, since most of the included studies had samples with less than 45 patients, which may involve a significant risk of bias [51]; furthermore, two studies had mixed diagnoses in their samples that may have led to a high risk of bias.

## 5. Conclusions

In conclusion, few functional neuroimaging studies currently concentrate on dissociative disorders. Nevertheless, it is possible to underline some consistent evidence on the cerebral functioning correlates of these diseases. Prefrontal cortex dysfunction seems prominent and can correlate with both traumatic identity states and neutral identity states in DID, and with the phenomena of depersonalization in DPD. In addition, changes in the functional neural network of the caudate were related to alterations of identity state and maintenance of an altered mental status in DID patients. Another role in DID seems to be played by a dysfunction of the anterior cingulate gyrus. Other areas involved in proprioception, interoception, self-awareness, and theory of mind, including parietal, temporal, and insular cortices, were reported to be dysfunctional in dissociative disorders. Further studies are needed to clarify the neurofunctional correlates of single dissociative disorders in affected patients, in order to identify better tailored treatments.

## Figures and Tables

**Table 1:** Main characteristics and results of functional neuroimaging studies on dissociative disorders.

Article	Tech	Participants	Age	Medications	Comorbidity	General Findings
Brand et al., 2009	PET	-Patients: 14 (11 males and 4 females) with Dissociative Amnesia (DSM-IV)	-Patients’ mean age: 36.57 years		-1 headache, migraine	Compared to controls, dissociative patients showed a significantly decreased glucose utilization in the right inferolateral prefrontal cortex.
Functional brain imaging in 14 patients with dissociative amnesia reveals right inferolateral prefrontal hypometabolism		-Control group: 19 healthy individuals (13 males and 6 females) without neurological or psychiatric history	-Controls’ mean age: 45.32 years		-1 closed head injury with headache, ataxia, right arm hemiparesis, diagnosis of conversion hysteria, diffuse abdominal and chest pain without organic causation	
					-1 motor symptoms (potential mild apoplexy)	
					-1 hypothyroidism	
Elzinga et al., 2007	fMRI	All participants were female	-Patients’ mean age: 40.8 (±10.7)	-1 mirtazapine (30 mg/day) and venlafaxine (75 mg/day)	Patients’ co-morbid diagnoses:	Both patients and controls activated brain regions involved in working memory (anterior, dorsolateral, and ventrolateral prefrontal cortex and parietal cortex).
		-Patients: 7 diagnosed with DID; 6 diagnosed with Dissociative Disorder NOS (DSM-IV, SCID-D)				
Neural correlates of enhanced working-memory performance in dissociative disorder: a functional MRI study.		-Controls: 16 healthy subjects without psychiatric disorder history	-Controls’ mean age: 34.6 (±10.9)	-1 citalopram 70 mg/day	-12 PTSD	Dissociative patients showed more engagement of left anterior prefrontal cortex, dorsolateral prefrontal cortex, and parietal cortex.
		RoB: high		All other participants were free of medication	-2 Major depressive Disorder	
		Possible cause of heterogeneity: two different diagnoses in the study sample			-4 Dysthymia	
					Patients’ history:	
					-10 Major depressive disorder	
					-6 Alcohol abuse	
					-3 Substance abuse	
Ketay et al., 2014	fMRI	-9 participants with Depersonalization Disorder diagnosis	Participants’ age: 18–55 years		Participants were medically and neurologically healthy subjects, besides their psychiatric diagnosis	Compared to controls, dissociative patients showed significantly more activation in right anterior cingulate cortex, bilateral medial prefrontal cortex, and left middle frontal gyrus in response to self vs. stranger faces.
Face processing in depersonalization: An fMRI study of the unfamiliar self		(SCID-I/P, SCID-II, SCID-D)				
		-10 control participants (Dissociative Experiences Scale’s score < 10)				
Lemche et al., 2007	fMRI	-9 patients (4 female, 5 male) with Depersonalization diagnosis (DSM-IV criteria)	-Patients: 36.1 ± 2.3 years	-3 paroxetine, fluoxetine, olanzapine (medicated with lowest effective doses)	6 patients had a secondary comorbidity of anxiety or depression (psychotic symptoms had been excluded)	When exposed to happy and sad facial expressions, patients showed lower subcortical limbic activity and negative correlations between skin conductance measures in bilateral dorsal prefrontal cortices.
Limbic and prefrontal responses to facial emotion expressions in depersonalization		-12 healthy subjects (5 female, 7 male)	-Controls: 27.3 ± 1.9 years	- 6 no medication		
Lemche et al., 2016	fMRI	-9 patients with diagnosis of Depersonalization Derealization Disorder	-Patients: mean age 36.11 (±2.34) years			Patients showed slight neuropsychological deficits in terms of reduced short-term memory, distractibility, and inability to suppress stress-related arousal states under cognitive task, but selective attention, cognitive inhibition and working memory were not impaired overall.
Cognitive load and autonomic response patterns under negative priming demand in depersonalization derealization disorder		(DSM-V criteria)				
		-12 healthy control subjects	-Controls: mean age 27.25 (±1.95) years			
Medford et al., 2006	fMRI	-10 patients (9 male, 1 female with diagnosis of Depersonalization Disorder (DSM-IV criteria)	Patients: 23–50 years (mean age 31.2, SD = 9.3)	-2 fluoxetine (40 mg/day)		Dissociative patients showed stronger recognition for clearly emotive words, but not for neutral words encoded in an emotive context. Furthermore, patients did not show activation of emotional processing areas during encoding process.
Emotional memory in depersonalization disorder: A functional MRI study		-12 healthy male controls	Controls: 22–34 years (mean age 27.8, SD = 3.6)	-3 lamotrigine (250 mg/day)		
				-1 lamotrigine (50 mg/day) and paroxetine (40 mg/day)		
				-4 no medication		
Reinders et al., 2006	PET	11 female patients with DID (according to DSM-IV criteria and SCID-D)	Patients’ age: 27–48 years		Exclusion criteria: pregnancy, traumatic experiences in a hospital setting, systemic or neurological illness, and no command of the Dutch language	PET data revealed different neural networks to be associated with different
Psychobiological Characteristics of Dissociative Identity Disorder: A Symptom Provocation Study						processing of the neutral and trauma-related memory script by neutral-related state and trauma-related state.
Reinders et al., 2014	PET	-11 patients (all female) with diagnosis of DID (DSM-IV, SCID-D)	-Patients’ mean age = 41.0, SD = 6.1)		Exclusion criteria: pregnancy, traumatic experiences in a hospital setting, systemic or neurological illness, and no command of the Dutch language.	DID patients showed similar pattern of activation to PTSD model.
Opposite brain emotion-regulation patterns in identity states of dissociative identity disorder: A PET study and neurobiological model		-16 healthy control subjects (all female) without psychiatric disease or current or past trauma-related problems, instructed to simulate dissociative identity states	-Controls’ mean age = 41.1, SD = 10.7)			The hypo-aroused identity
						state activates the prefrontal cortex, cingulate, posterior association areas, and parahippocampal gyri (overmodulating emotion regulation), whereas the hyper-aroused identity state activates the amygdala,
						the insula, and the dorsal striatum, (undermodulating emotion regulation).
Reinders et al., 2016	PET	29 subjects:	-Patients: mean age 41.0 (SD = 6.1)			Prefrontal hyperactivation during neutral personality state in response to trauma-related words vs. neutral words.
The Psychobiology of Authentic and Simulated Dissociative Personality States: The Full Monty		-11 patients with DID (DSM-IV)				Right hippocampus and left amygdala different activation patterns have been associated with DID.
		-18 mentally healthy control individuals (all female): 10 high-fantasy-prone DID-simulating, 8 low-fantasy-prone DID-simulating (without history potentially traumatizing events)	-High-fantasy-prone DID-simulating controls: mean age 38.2 (SD = 10.9)			Involvement of the caudate nucleus both in the switch between identity states and in the maintenance of the altered identity state.
		Controls were instructed to simulate different dissociative personality states and write the autobiographical analog “neutral” and “trauma” memory scripts.	-Low-fantasy-prone DID-simulating controls: mean age 42.5 (SD = 10.1)			
Schlumpf et al., 2013	fMRI	-Patients: 15 females with Dissociative Identity Disorder diagnosis (DSM-IV criteria and SCID-D), considering the ANP and EP prototypes of DID.	-DID group mean age: 43.3 years (SD = 9.1)	-13 patients: mainly antidepressant	Exclusion criteria:	Compared to DID-ANP, DID-EP showed longest reaction times when exposed to neutral faces and more involvement of the parahippocampal gyrus.
Dissociative part-dependent biopsychosocial reactions to backward masked angry and neutral faces: An fMRI study of dissociative identity disorder.		-DID simulating control group: 15 female actors, who were instructed to simulate DID-ANP and DID-EP.	-Simulating control group mean age: 43.2 years (SD = 10.4)	-2 patients: no medication	-comorbid psychosis, drug abuse or addiction, antisocial or histrionic personality disorder, neurological or organic brain disease	When compared to EP-simulating subjects, DID-EP showed more involvement in the brainstem, face-sensitive regions, and motor-related areas.
						DID-ENP, when exposed to neutral and angry faces, showed a decreased activation all over the brain.
Schlumpf et al., 2014	fMRI	-Patients: 15 females with Dissociative Identity Disorder diagnosis (DSM-IV criteria and SCID-D), considering the ANP and EP prototypes of DID.	-DID group mean age: 43.3 years (SD =9.1)	-13 patients: mainly antidepressant	Exclusion criteria: comorbid psychosis, drug abuse or addiction, antisocial or histrionic personality disorder, neurological or organic brain disease	ANP-EP comparison: ANP perfusion patterns showed elevated involvement of bilateral thalamus, EP showed increased perfusion in the dorsomedial prefrontal cortex, primary somatosensory cortex, and motor-related areas.
Dissociative Part-Dependent Resting-State Activity in Dissociative Identity Disorder: A Controlled fMRI Perfusion Study		-DID simulating control group: 15 female actors, who were instructed to simulate DID-ANP and DID-EP.	-Simulating control group mean age: 43.2 years (SD = 10.4)	-2 patients: no medication		ANP and EP- simulating controls fitted their role-play strategies, activating brain structures involved in visual mental images and empathizing feelings.
			(The sample was part of a larger study)			
Vissia et al., 2022	fMRI	62 female participants:	The sample was part of a larger multicenter study			Results showed the activation of prefrontal parietal network, main working memory in the left frontal pole and ventrolateral prefrontal cortex in all three simulated neutral states and in trauma-related identity states of DID-simulators, but not in trauma-related identity state of DID-patients and PTSD-patients, which did not activate parietal regions.
Dissociative identity state-dependent working memory in dissociative identity disorder: a controlled functional magnetic resonance imaging study		-14 patients diagnosed with Dissociative Identity Disorder				
		-16 DID-simulating healthy controls				
		-a paired of control group: 16 subjects with PTSD and 16 healthy controls (control group consisting of NIS and TIS)				
Weniger et al., 2013	fMRI	-Patients: 14 female inpatients with history of childhood abuse and a diagnoses of Dissociative Amnesia or DID (DSM-IV, SCID-D)	-Patients’ age: 24–50 years	-7 patients were medicated with antidepressant: doxepine, mirtazapine, fluoxetine, paroxetine, venlafaxine, duloxetine	-10 Dissociative Amnesia	Compared to controls, dissociative patients showed a similar (although weaker) pattern of activity changes during egocentric learning. Dissociative disorder severity was associated to better performance and to stronger activity in cingulate gyrus and precuneus. Attentional and visuospatial mnemonic functioning were preserved in individuals with dissociative disorder.
Egocentric virtual maze learning in adult survivors of childhood abuse with dissociative disorders: Evidence from functional magnetic resonance imaging		-Controls: 14 healthy females (no neurological or psychiatric history, no traumatic exposure history)	-Controls’ age:	-4 patients were medicated with antipsychotics (aripiprazole, quetiapine)	-10 DID	
		RoB: high	21–48 years	-3 patients were free of psychotropic medications	-4 Depersonalization Disorder	
		Possible cause of heterogeneity: two different diagnoses in the study sample			-12 Borderline Personality Disorder	
					-6 Major Depression	
					-9 Anxiety Disorder	
					Exclusion criteria: history of PTSD, psychotic disorder, neurological disorder, current substance use	

*Legend*. ANP: Apparently Normal Part; DID: Dissociative Identity Disorder; DSM: Diagnostic and Statistical Manual of mental disorders; EP: Emotional Part; fMRI: functional Magnetic Resonance Imaging; NIS: neutral identity state; NOS: Not Otherwise Specified; PET: Positron Emission Tomography; PTSD: Post-Traumatic Stress Disorder; RoB: Risk of Bias; SCID: Structured Clinical Interview for DSM; TIS: trauma-related identity state.

## Data Availability

Not applicable.

## Supplementary Materials

The following supporting information can be downloaded at: https://www.mdpi.com/article/10.3390/jpm12091405/s1. Table S1: PRISMA 2020 checklist.
